# “Something Good Has to Come Out of the Horror”: A Qualitative Examination of Cancer Survivors' Attitudes Towards Participation in Research During the First Year of the COVID-19 Pandemic

**DOI:** 10.3389/fpubh.2021.741188

**Published:** 2021-10-29

**Authors:** Louis Fox, Harriet Wylie, Anna Haire, Saran Green, Joyce Kibaru, Mieke Van Hemelrijck

**Affiliations:** Translational Oncology and Urology Research, School of Cancer and Pharmaceutical Sciences, King's College London, London, United Kingdom

**Keywords:** cancer, COVID-19, recruitment, accrual, participation, pandemic, epidemic, behaviour

## Abstract

**Introduction:** The first year of the COVID-19 pandemic has been highly disruptive for people with cancer. Furthermore, it has been shown that accrual to cancer trials dropped substantially in 2020. Building on findings from a previous pilot survey, the present study used qualitative methods to gain insights into attitudes towards participation in research studies amongst people who have experienced cancer, in the context of the first year of the COVID-19 pandemic.

**Materials and Methods:** We interviewed 13 participants from the UK, who were purposively sampled, including a broad sample of cancer types, and a mixture of individuals who have and have not taken part in research previously. Participants underwent semi-structured interviews (median interview duration: 47 min) and were asked open-ended questions about their attitude towards and experiences with COVID-19, and their attitude towards research participation. In addition to this, prompts were used to ask participants about concerns that were highlighted by our previous quantitative work on this topic, such as concerns about being older or having to travel to participate. Interview transcripts were analysed using a framework analysis approach.

**Results:** Our findings suggest that cancer patient decision-making about research participation during an infectious disease pandemic may be a function of a basic cost-benefit analysis, which considers the benefit of taking part, either personally to themselves or to wider society. The benefit may then be weighed by the patient against the risk of being infected, which may be influenced by trust in the relevant clinicians/researchers; familiarity with the study location; provision of detailed information on safety protocols for infectious disease; and, in particular, the availability of safe transport to and from the study location.

**Discussion:** Some cancer patients say that they would be less likely to participate in a research study in the middle of an infectious disease pandemic due to an increased risk to themselves. Patients' perceived risk to themselves from participating may be ameliorated *via* the provision of certain practical solutions that can be considered at the study protocol design stage, such as safe travel, information, and the use of staff and study sites familiar to the patient.

## Introduction

On 11th March 2020, the World Health Organisation declared the emergence and outbreak of the novel coronavirus SARS-CoV-2 a pandemic (1). The worldwide outbreaks of the virus and its related disease, Coronavirus Disease 2019 (COVID-19), have been hugely disruptive to healthcare systems in many countries, affecting both the delivery of services, and healthcare utilisation (2–4). Many cancer clinical trials were suspended at the onset of the pandemic (5), and other cancer-related studies have also experienced difficulties (Fox et al.^1^).

The initial onset of the pandemic was followed by large decreases in accrual rates to clinical trials, with some reports from the United States indicating decreases of around one half (6, 7). While much of this decrease is attributable to the suspension of operations, there remains a concern that the pandemic has created hesitancy in some individuals to participate in research studies, which can often require additional commitments from a patient (8, 9). The pandemic is likely to pose a substantial healthcare concern for years to come. Given the various challenges faced by the field of cancer research as we move through the pandemic [Fox et al.^1^; (10)], it is important that participant accrual to research studies is maximised to its full potential. Such an endeavour is important because there is a need to maximise value from available resources, which appear to have been strongly impacted by the pandemic (11). Furthermore, if the social disparities in health that have received a renewed focus in 2020 (Fox et al.^1^) are to be addressed, there is a need for strategies to maximise participation from socially disadvantaged groups (12), particularly minority ethnic individuals, who are at an increased risk from both COVID-19 and cancer, both in terms of incidence and outcomes (12, 13).

Existing research has identified numerous factors that may influence motivation to take part in research studies in people with cancer. Broadly speaking, in the existing literature, pertinent factors have been identified as spanning the domains of “patient-level,” “physician-level,” and “system-level” factors (14–16). Here we focus on “patient-level” factors, as this is the category that encompasses patient attitudes, which are the focus of the present study. A recent review and meta-synthesis of qualitative studies examining cancer patients' processes of deciding to enrol in a clinical trial was conducted by Viljoen et al. (15). Forty studies were included in this review, of which the publication years ranged from 1999 to 2018. The reviewers found that in deciding whether to take part, patients undertook a risk-to-benefit appraisal of participation, that was influenced by numerous factors. This observation is consistent with a previous review conducted in 2010 of decision-making processes in this setting (17). In Viljoen et al.'s qualitative review, such factors included: (1) the patient's degree of trust and confidence in their healthcare professionals; (2) the style of communication of information about the trial between physician and patient (which, when suboptimal or rushed, could lead to patients feeling uninformed and alienated); (3) the sense of moral obligation, or altruism, towards society; and (4) the reassurance that they will be adequately cared for during study procedures. Further patient-level factors that may influence risk-to-benefit appraisal in this setting have been recently documented by other investigators, and these include a fear of treatment side-effects; disliking the idea of being experimented upon; issues or complications arising from travel to the study site; burden of study procedures (including emotional burden); knowledge about the study/intervention; concerns about the physical setting of the study; and physician's attitude towards the study (14, 16, 18). It is conceivable that patients' considerations related to the COVID-19 pandemic may modify their experience, or appraisal, of several of these identified factors. For example, concerns about whether the physical setting of the study will keep them safe from SARS-CoV-2 infection, or concerns about exposure to SARS-CoV-2 during travel to the study site.

Considering these issues, we aimed to identify potential sources of hesitancy to take part in research amongst people with cancer, that have been precipitated or exacerbated by the COVID-19 pandemic. Our objective was to use qualitative methods to: (1) explore reasons why individuals with cancer may, or may not, be motivated to participate in research during the pandemic; and (2) use this information to identify potential strategies for minimising hesitancy to participate in research during the pandemic. Preliminary work was undertaken by our group in the form of a small online survey (9), to identify potential issues that may need to be explored in the present, qualitative study. The findings of that preliminary work indicated that general anxiety, concerns about cancer, and having to travel to participate were likely to be factors in likelihood of research participation amidst the COVID-19 pandemic. These findings informed the approach of the present study.

This study was approved by the Research Ethics Office at King's College London (LRS-19/20-19677).

## Materials and Methods

Study methods and results are reported in line with the Consolidated Criteria for Reporting Qualitative Research (COREQ) checklist (19).

### Study Design and Participants

The study was an interview study, utilising a framework analysis approach. A framework analysis approach was used because this analytical approach is well-suited to examining multiple aspects of a practical problem and how these different dimensions might be related, hence providing indications of practical solutions. For this reason, it has been used previously by investigators to examine factors influencing recruitment to health research studies [e.g., (20, 21)]. We conducted 1:1 semi-structured interviews with 13 individuals who had received a cancer diagnosis previously, of various cancer types (see Table 1). Participants were recruited purposively, in accordance with the aim of ensuring that cancer types that are perceived have particular relevance to COVID-19 (e.g., lung, blood) were represented, as well as individuals who both had, and had not, participated in a cancer research study before. These sampling strategy criteria were informed by results of the preliminary survey described above. Participants were approached either *via* social media with the co-operation of patient organisations/charities (i.e., participants responded to an advert posted in social media by a patient organisation/charity); or *via* cancer patient organisations/charities themselves, who approached participants *via* email on our behalf. Two potential participants who were approached declined to participate (in the form of non-response).

**Table 1:** Characteristics of interviewees.

**Participant no**.	**Age**	**Sex**	**Cancer type**	**Most recent treatment**	**Taken part in cancer research before?**
P1	60-69	Female	Breast	Chemotherapy	Yes (previously)
P2	60-69	Male	Prostate	Hormone therapy	Yes (previously)
P3	60-69	Female	Bone marrow	Chemotherapy	Never
P4	30-39	Female	Blood	Chemotherapy	Never
P5	60-69	Male	Lung	Chemoradiotherapy	Never
P6	50-59	Female	Ovarian	Other	Never
P7	50-59	Female	Non-Hodgkin's lymphoma	Immunotherapy	Yes (previously)
P8	50-59	Female	Breast	Hormone therapy	Yes (previously)
P9	60-69	Female	Colorectal	Chemotherapy	Yes (currently)
P10	70-79	Male	Prostate	Radiotherapy	Yes (previously)
P11	60-69	Male	Prostate	Surgery	Yes (previously)
P12	50-59	Female	Blood	Chemotherapy	Never
P13	50-59	Female	Kidney	Immunotherapy	Yes (currently)

### Interviewer and Participant Characteristics

Interviews were conducted by LF (he/him), HW (she/her), AH (she/her), and SG (she/her), after a demonstration and training/Q&A session was provided by LF, who is a PhD-level research associate and an experienced qualitative researcher. HW and AH are both clinical trials co-ordinators who have extensive patient-facing work experience delivering clinical studies in cancer. SG has extensive work experience of co-ordinating patient and public involvement activity for cancer research. Hence all interviewers were considered to possess the appropriate interpersonal skills to be able to conduct the interviews, following the training session.

Interviewers did not know the participants they were interviewing, apart from one (P1), who was a former colleague. The interview with P1 was performed before any others and used as a training session, and to pilot the topic guide. The interview with P1 was included in the analysis, due to valuable insights gained during this interview. All participants were made aware of the reasons for doing the research prior to their interview, but were not told the specific topics that we were interested in. Interviewers were cognisant to explore specific topics that had emerged in previous preliminary work; but were also guided by a topic guide that ensured that a comprehensive set of topics were covered.

### Interviews

Each participant underwent a single interview (median duration: 47 min, range: 13–67 min), conducted *via* video call software due to COVID-19 safety measures (i.e., from the participants' home). Participants' family members were occasionally present in the home but did not take part in interviews. A pilot tested topic guide (which was partially based on observations from a previous preliminary survey, referenced above) was used by researchers. Participants were asked about their background and general attitude towards research. Then, they were asked questions to establish their attitude to the pandemic in general, such as “*Are you worried about catching COVID-19?”* Next, they were asked questions about research participation during the pandemic, such as “*How would you feel about participating in a study that involved repeated hospital visits?*” and “*What would make you feel more comfortable to participate in a research study based at the hospital, whilst the COVID-19 pandemic is ongoing?*” Questions were also asked about different types of studies, and probes were used to investigate issues that seemed to be of particular concern to participants. Interviews were audio recorded and were transcribed by a transcription service. Returned transcriptions were checked against recorded audio for quality assurance. No field notes were taken. Transcriptions were not returned to participants for member checking. It was established *a priori* to conduct a minimum of 10 interviews and then cease interviews when no new themes emerged from three successive interviews after that. As no new themes emerged in the final three interviews analysed, interviewing was ceased after 13 interviews.

### Analysis

Interview transcripts were analysed primarily by LF using a framework analysis method (22), with sense checking of the data interpretation by HW, AH, and SG, who had conducted the interviews. NVivo 12.0 was used to assist the analysis. The research question for the analysis was: *What factors that are related to the COVID-19 pandemic might make an individual either more or less likely to participate in a cancer research study?* LF first familiarised themselves with the data by reading and re-reading all transcripts and taking notes. As a result of this process, a draft theoretical framework was created that encompasses the main ideas and themes. The raw data were then “indexed,” i.e., reprocessed against this framework, to fine tune the framework by ensuring that the raw data were a good fit. The indexing process resulted in the creation of some additional categories in the framework. The data were then “charted” in a spreadsheet, applying the framework across the X axis and individual participants on the Y axis. All charted data were reduced into easily discernible pieces of information, so that the large amount of textual data could be synthesised in a process of physical mapping and interpretation (see Supplementary Material). These processes were all undertaken independently by LF. For cross-validation, the reduced charted data were provided to the other interviewers (HW, AH, and SG) to check that the interpretations of the transcripts were consistent with their interview experience. There were no inconsistencies raised as a result of this. The process of mapping and interpretation allowed for different types of perspectives, observations, or participant characteristics to be cross-referenced by LF, and transformed into a conceptual map of the key insights and how these related to one another. The key insights and relationships between concepts were sense-checked by HW, AH, and SG, and are presented in a structured narrative format below, in accordance with the structure of the overall findings. Findings were not returned to participants for feedback.

## Results

**Figure 1:**
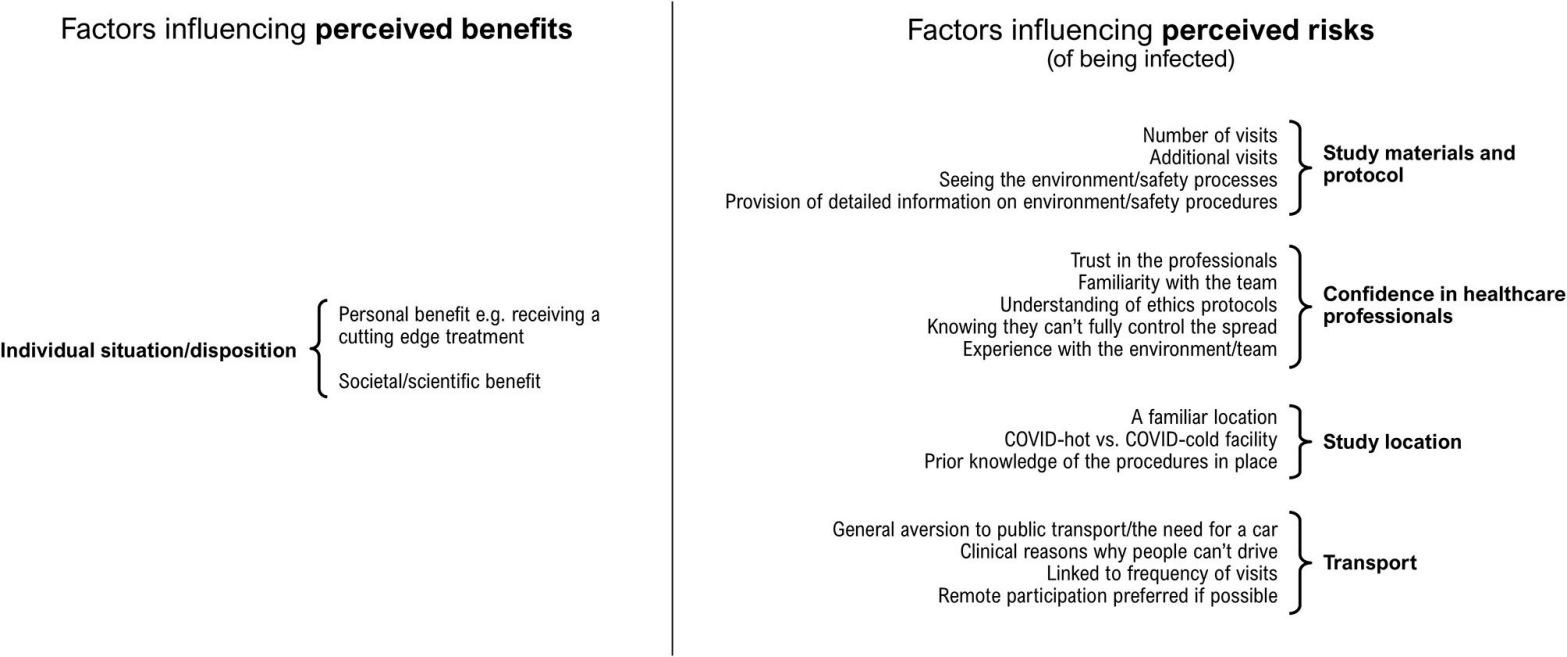
Factors that were associated with participants' perceptions of costs, or perceptions of benefits, of participating in a cancer research study during 2020, the first year of the COVID-19 pandemic.

Most participants indicated that they would probably be happy to participate in a research study despite the pandemic. However, it was clear that there were several factors that could influence an individual's decision whether to take part. Insight gained from the analysis showed that in our sample, participants' attitudes towards cancer research participation in the context of the COVID-19 pandemic were consistent with a basic “cost-benefit” analysis, i.e., contingent on a balanced appraisal of (1) the perceived risk of being infected that may be produced by participation; against (2) the potential perceived benefits of participation (see Figure 1).

### Perceived Risk of Being Infected

Factors that might influence our participants' perception of the risk of being infected were characterised by four focal areas: (1) confidence in healthcare professionals; (2) study materials and protocol; (3) study location; (4) public transport.

#### Confidence in Healthcare Professionals

Many research protocols involve hospital visits. Participants' views about the safety of the hospital environment varied. For example, one participant said that they “*think of the cancer centre as a safe haven, because I don't feel like they're going to put me at risk at all, because of their protocol”* [P2, male, metastatic prostate cancer]. Another stated that they thought that supermarkets are “*far worse than the hospital”* [P9, female, colorectal cancer].

In contrast, other participants were unconvinced about safety, citing their experiences of being in hospital during the pandemic. One participant said that they “*observed plenty of opportunity for transmission of the virus”* in hospital [P13, female, metastatic kidney cancer]. Another said that “*you don't feel so safe there”*, as they were “*walking in very close proximity to other patients and loads of staff, porters, cleaners and stuff like that”* [P11, male, prostate cancer]. Healthcare staff were not held responsible by these participants quoted, however:

“*The staff are doing everything they can, but there are still other people walking around … I know they're meant to clean everything and all that, but I can only say there wasn't 100% effort going into that, borne out of necessity because they were so busy.”* [P11, male, prostate cancer]

“*[The hospital staff] were under a lot of time pressure, which is why I don't think they had time to do that sanitising or not be going to a COVID patient. I know my guy was going to a COVID patient next.”* [P13, female, metastatic kidney cancer]

But another participant thought that some healthcare teams were better than others:

“*Some people clean an environment really, really well, and they leave a gap of half an hour between each patient and make sure that there is a proper team that cleans it properly, blah, blah. Others, I think it's not so good.”* [P6, female, ovarian cancer]

However, in general most of the participants expressed the sentiment that although nowhere could be absolutely safe, some pragmatic level of risk was acceptable when visiting hospital:

“*You can't possibly have 100% control over [the virus spreading], so you just have to go with the best that you can.”* [P12, female, acute lymphoblastic leukaemia]

“*Generally, it felt okay … [the hospital staff] were really good about it, really professional … it feels like they have adapted really well to keeping you as safe as possible. Obviously, there are still risks.”* [P4, female, acute myeloid leukaemia]

Some participants indicated that their experience of cancer care had instilled deep trust in their healthcare team, which gave them reassurance:

“*I've got total, total confidence in the people that look after me. I just know I'd put my life in their hands. And if their decision was that I needed to go up to the cancer centre, I'd go up there, and I'd feel safe.”* [P2, male, metastatic prostate cancer]

“*I've got immense faith in them. They saved my life.”* [P12, female, acute lymphoblastic leukaemia]

Another participant made the point that there are a lot of things in life in which they trust their personal safety to professionals:

“*There are a lot of things that we take part in in life and we have to put our hands in the people [sic] who are in the knowledgeable position of setting them up. That's it really, you just have to believe in them and go for it, yes?”* [P10, male, prostate cancer]

It was noted by a participant that their experience of visiting hospital and seeing the safety protocols first-hand had made them less anxious about visiting afterward:

“*I got a lot more confidence … I was very happy with what I saw actually happening in the hospital, and how much care was being taken … I think that's a problem for a lot of people who haven't been into a hospital, that initial anxiety. If they once went into, they'd probably feel, ‘Oh, it's not so bad after all.' Yes.”* [P9, female, colorectal cancer]

In summary, there were encouraging signs amongst the participants that they trusted their healthcare teams to safeguard their well-being, with much of this trust emerging from positive experiences of cancer care.

#### Study Materials and Protocol

Participants made comments about some factors that are borne out of study protocols. Some (although not all) participants expressed concerns about having to undertake additional visits to hospital purely for a research study.

“*If there were extra trips to the hospital that were not potentially of clinical benefit to me, then I would find that quite hard to justify at the moment, I think.”* [P4, female, acute myeloid leukaemia]

“*I think you would have to be able to reassure me that I was going into a completely sterile, cleaned environment with no risk getting there. Because why would anyone put themselves at risk during this time when you're already vulnerable? It wouldn't make any sense.”* [P6, female, ovarian cancer]

The number of visits required was also cited as a potential concern:

“*If they were asking me to go once every few months, that's one thing. If they were asking me to go every week, or every day, then that is something different again, isn't it?”* [P12, female, acute lymphoblastic leukaemia]

Some participants indicated that their willingness to partake in additional research-only visits would be conditional on the study location and availability of safe transport, which are elaborated on in the relevant sections below.

It was noted by participants that being provided with detailed information on the safety protocols in place with regards to COVID-19 would be reassuring and would encourage them to participate:

“*You know, are the rooms cleaned and will they be wearing PPE or at least a mask? That sort of thing, what exactly they're going to do, I would want to know that.”* [P1, female, breast cancer]

“*I think you'd just want to know about the procedures if you're having to go into hospital. Exactly what are the procedures? Where is your temperature taken, or would the number of people who you interact with be minimised?”* [P3, female, multiple myeloma]

A further participant commented that they would “*like to get there and have a look around”* [P11, male, prostate cancer]. It was suggested that information on safety protocols could be given to potential participants in the form of a leaflet to supplement the other study information, which “*showed you what you had to do … I think that's quite important”* [P1, female, breast cancer].

In summary, some participants indicated that the provision of detailed information on how COVID-19 safety protocols are being implemented would be a reassuring factor that may influence likelihood of participation. It was also apparent that, for some participants, the number/frequency of additional hospital visits would need to be weighed up against other factors when deciding whether to participate; and that protocols allowing for research appointments to coincide with clinical appointments would be the most attractive:

“*You might find people will engage better if it happens to coincide … it's almost a frustrating – because you're going in for a five-minute blood test and it takes me an hour and a half to get there. So, actually, if there is something else to do when you're there… (Laughter)”* [P6, female, ovarian cancer]

#### Study Location

It was important for some participants to have confidence in the facility that they would be visiting for research. For some, this was about familiarity:

“*I think it would depend what it was, at which hospital … I'm fairly confident, when I go into my hospital- Well, not confident, but I feel I know what happens there. So, when I have to go to a different hospital, I get a bit more anxious.”* [P6, female, ovarian cancer]

“*I think it probably depends on the hospital. But absolutely 100% at [participant's oncology hospital]. If you took me to [general hospital], I would be a bit more nervous.”* [P12, female, acute lymphoblastic leukaemia]

As noted above, in some participants this attitude also seemed to be produced by first-hand experience of safety protocols in place at their hospital.

“*Going to [participant's usual hospital] for a blood test, you have to go to a desk at the door and they give you a new mask, they make you wash your hands. You know, everything is geared up. And they show you where to stand. It's fantastic, you feel safe.”* [P2, male, metastatic prostate cancer]

There was also a reluctance to visit a hospital in some patients based on whether the hospital was ‘COVID-hot':

“*If I had to, for example, go to [general hospital], where there is COVID. If I had to go there every week for tests, then I would say, ‘No, I'm not doing it,”'* [P3, female, multiple myeloma]

In summary, participants seemed to indicate that the COVID-19 pandemic had made them less amenable to visiting an unfamiliar location for a research study (particularly a “COVID-hot” centre), as outlined clearly by the below quote:

Interviewer: “*If there was a research project which involved quite a few number of extra visits, would that be something that would concern you during this time?”*

Respondent: “*No. Not extra visits to my own hospital, no. I wouldn't worry about that. Extra visits to other hospitals, yes.”* [P3, female, multiple myeloma]

#### Transport

The use of public transport to visit a research site emerged clearly as the most frequently cited and emphasised issue amongst the participants. Many participants were very hesitant (or simply refused) to use public transport. Although one participant said that they “*would certainly have no problem with getting on a bus or a train”* [P10, male, prostate cancer], almost all other participants cited significant concerns about taking public transport during the pandemic:

“*I would definitely think twice about it … the public transport was probably a greater risk than being in a hospital I would have thought.”* [P11, male, prostate cancer]

“*I wouldn't do it without [safe transport], because why would I? It wouldn't make sense.”* [P7, female, non-Hodgkin's lymphoma]

It emerged that for some participants, their willingness to participate in hospital visits for research was fully contingent on car access:

“*I would be happy to [participate]. I would drive up, I don't think I'd go on public transport. I don't think I'd be confident enough to be able to protect myself from other people … once you get to the cancer centre, no problems at all, it would be the travelling.”* [P2, male, metastatic prostate cancer]

Interviewer: “*How do you get there?”*

Respondent: “*I drive.”*

Interviewer: “*You drive, okay. Public transport, would that be something you'd consider?”*

Respondent: “*No, not at all.”*

Interviewer: “*No. Because of COVID, is that?”*

Respondent: “*Because of COVID.”* [P3, female, multiple myeloma]

Moreover, one participant with metastatic kidney cancer informed us that because of a brain metastasis—and subsequent radiotherapy—they were unable to drive and therefore would not be keen to participate:

“*I had radiotherapy last year on my brain [metastasis] as well, I'm not allowed to drive anymore. So, to get to any community hub would involve using at least two forms of public transport, which I wouldn't be keen to do.”* [P13, female, metastatic kidney cancer]

A small number of our participants were interviewed after receiving their first dose of vaccination against SARS-CoV-2, which appeared to make one participant more amenable to taking public transport, but not totally unconcerned:

“*The vaccine has helped … but I still wouldn't go on a bus … I would perhaps go on a train, now I've had the vaccine, yes.”* [P9, female, colorectal cancer]

Remote research—where feasible—was deemed by participants to be acceptable and for the large part preferable. It was also suggested by a participant that in-person research appointments could be scheduled for off-peak times to prevent participants having to take crowded transport.

“*Yes. I think that that [off-peak appointments] could be really important because public transport, it does vary how crowded it is at different times of day, or, you know, if people don't have their own car.”* [P9, female, colorectal cancer]

In summary, getting public transport was a substantial concern amongst the participants, appearing to be more so of a concern than attending the hospital *per se*. Participants who had access to a car indicated that if this were not the case, they would be unlikely to participate in a study that involved in-person visits during the pandemic.

### Perceived Benefits of Participating

As outlined above, many participants appeared to be engaging in a basic cost-benefit analysis to appraise whether they might participate in a study, balancing risks against potential benefits—either to themselves or society.

“*If there were extra trips to the hospital that were not potentially of clinical benefit to me, then I would find that quite hard to justify at the moment, I think.”* [P4, female, acute myeloid leukaemia]

“*I know that by doing research, that's the only way to improve things in terms of cancer. So, yes, I might be slightly concerned, but it wouldn't put me off going into participating [in] research.”* [P9, female, colorectal cancer]

In terms of the perceived benefits, a small number participants were focused on benefits to themselves personally:

Interviewer: “*If you were offered a trial … which wouldn't really benefit you but it might help others, is that something you would consider in the current climate?*

Respondent: “*To be honest, I would take my own health first.”* [P2, male, metastatic prostate cancer]

“*Of course, it depends what the research is. So, if it's a research [sic] that I feel actually might be beneficial to me, because I have incurable cancer, so I'm fighting for my life every day, then I might [take part].”* [P6, female, ovarian cancer]

However, most of the participants stated the importance for future patients, or “paying something back,” as a main driver for wanting to participate in research, despite the perceived risks:

“*I am delighted with the results I have had from the NHS and I thought, ‘If I can help somebody else, I'm happy to do that.' A bit like paying back really, in a way.”* [P5, male, lung cancer]

“*I would more than likely participate, because if it stops other people going through that hell, then it's worth it, frankly. I've always had this view that something good has got to come out the horror.”* [P12, female, acute lymphoblastic leukaemia]

For some participants, the relative scientific value of the project was seen as crucial to their willingness to participate during the pandemic…

“*I'd probably say no unless it was something I thought would be quite crucial for the good or others or for the good of myself.”* [P11, male, prostate cancer]

…including one participant who said that during the pandemic, they would feel more attracted to a study involving so-called “hard outcomes”:

“*If it was a matter of I'm considering doing something that puts someone else at risk, or put myself at risk, I think I would be more likely to do that if it was a drug trial. Because literally trading off your risk of dying against your risk of not dying, or helping people not die, whereas you're trading off life and death against the softer outcomes, that is- I don't really like to admit that, because I'm all in favour of soft outcomes and mental health and all that stuff. But I feel like we're still in that kind of crisis moment now, where actually you have to create a pecking order.”* [P4, female, acute myeloid leukaemia]

In summary, among the participants there were underlying motivations to take part in research which seemed to be somewhat resilient to wider events. However, such motivations varied between individuals, with some individuals more oriented towards personal benefits, and others more oriented towards societal benefits. There was also an indication that during the pandemic, the extent to which a study is perceived as “vital” may influence some participants' decisions to take part. Whether a study is perceived to be “vital” could potentially be influenced by the presence of so-called “hard” outcome such as survival, as opposed to so-called “soft” outcomes.

## Discussion

In our study many participants had an underlying motivation to continue to participate in research due to the perceived benefits, which appeared to vary as a function of the perceived priority—or overall scientific value—of the project, and for some, the personal benefits to themselves. When extracts were analysed in the framework matrix alongside the other analysis areas examining perceived risk, it was apparent that many participants were willing to accept a certain degree of risk to themselves to achieve these benefits. The degree of perceived risk inferred by participants was largely influenced by practical considerations such as mode of travel; being provided information on safety protocols in advance; the number of additional research-only visits required; and being able to attend a familiar (and ideally “COVID-cold”) centre.

There was an encouraging degree of trust in healthcare staff expressed by participants that seemed to translate into reassurance and goodwill, in terms of research participation. There were however some participants that were particularly anxious about the pandemic, who it seems were unwilling to accept any incremental risk to themselves produced by research participation. Public transport was viewed as inherently high-risk by participants, some of whom stated that having to take public transport to participate would be a “deal breaker” of sorts. The collective appraisal of all these issues by participants appeared to produce a basic cost-benefit appraisal which would inform their decision whether to participate.

The findings of this study of COVID-19 are consistent with many aspects of what has previously been reported in the literature. The previous meta-synthesis conducted by Viljoen et al. reported that cost-benefit appraisals by cancer patients were influenced by the degree of trust in their healthcare professionals, and whether they perceived that they would be adequately taken care of (15). Our findings in the context of COVID-19 were reflective of this, suggesting that perhaps to an extent, the nature of the risk itself may be subordinate to the degree of trust that individuals place in their healthcare professionals. Viljoen et al. also reported that a sense of altruism towards society was a key factor in motivating individuals to take part in research studies. This was also apparent from our investigation, in which for some participants, this sentiment seemed to be an overriding factor for some of the perceived risks (dependent on the perceived importance of the research). Further, results from our study are consistent with the finding of Viljoen et al. that many patients desire that their healthcare providers “offer detailed discussions and deliver general and specific information” about studies recruiting, to improve patients' understanding of the risk-to-benefit ratio (15) (p. 1276). We found that the provision of detailed information to patients about COVID-19 safety procedures embedded in study protocols may encourage patients to participate. Hence, our study provides some initial indications that previous findings in this area may be generalisable to the COVID-19 context.

Many of the factors considered here are determinable at the stage of procedural design for research studies. As such, they could be considered by study designers operating in an epidemic/pandemic scenario. For example, potential participants can be given detailed information on infectious disease safety protocols in advance of their decision, alongside their standard study information sheet. Study designers might consider the relative advantages of minimising in-person visits and/or ensuring that data collection time points coincide with standard clinical appointments. Remote participation could be offered where appropriate. Familiarity of the clinical/study team and the study centre to patients could be prioritised, alongside an awareness of which centres have the potential to be “hot” or “cold” in terms of catering to the pandemic. Where additional research visits are necessary, provisions for alternatives to public transport could be offered (ideally without participants having to cover costs up front).

One key barrier to participation that has been previously identified is travel concerns (16, 18). It is of concern that in our COVID-19-specific study, a crucial factor that seemed to be underlying participants' willingness to undertake in-person visits was car ownership, implying that the presence of COVID-19 may compound previously observed logistical issues related to transport. Importantly, the implications of hesitancy to use public transport to take part in research are likely to disproportionately effect disadvantaged communities, who would be expected to rely more on public transport. The resulting impact on sample diversity would further exacerbate an existing problem, given that people of colour and/or low socioeconomic status are already systematically underrepresented in cancer studies (23, 24). This key finding of our study highlights an intersectional issue, whereby the interaction between COVID-19, deprivation, and participation in cancer research could promote a future outcome in which an individual's ethnicity may be underrepresented by care practices, due to research participation barriers exacerbated by the COVID-19 pandemic. Intersectional health equality issues entwining ethnicity and deprivation have been shown previously to be linked to “hard” outcomes such as cancer survival [e.g. (25)], and there is indeed some evidence that the relationship between ethnicity and health is modified by car ownership (26). Our study did not possess the scope to explore such health inequality issues further, and we would suggest that this is a key area for future study. It is also important to note that, as reported by one of our participants, driving is contraindicated for individuals with certain clinical backgrounds such as brain metastases. Such observations underline the need for the provision of transport for participants, or the minimisation of in-person visits, in an infectious disease epidemic/pandemic scenario.

It was encouraging to see that in our study, many participants' trust in their healthcare teams was acting as a motivating factor for research participation during the pandemic. Patients' trust in the healthcare professionals and researchers conducting the study has been identified as a key determinant of consent to cancer research participation in many studies [e.g. (27–30)]. The importance of good relationships between patient and healthcare team, and of community networking, has been suggested to be crucial in maximising cancer research engagement, particularly amongst some ethnic groups (29, 30). When taken together with our findings, such studies suggest that maximising research participation in an epidemic/pandemic scenario may in part be influenced by who approaches patients about the research; the quality of the explanation of the research to the patient; and whether the research is undertaken locally, in a setting the patient is familiar with. Our findings indicate that a study that is presented favourably in this manner may reassure participants that they will not be exposed to a dangerous pathogen, as a matter of a lack of diligence on the part of the clinicians/researchers involved.

Participants in our study represented the view that the higher degree of “impact” they perceived the study to have might influence whether they were willing to overlook potential risks of taking part. One participant noted that they might be more likely to participate in a study including so-called “hard” outcome, such as survival, as opposed to so-called “soft” outcomes. We could not find any studies in cancer that have examined whether there is a difference in patients' attitudes towards participating in studies with so-called “hard” or “soft” outcomes. Such an incidental question may be worth examining, given the increasing rates of long-term survival in certain cancer types. In terms of the epidemic/pandemic context, our findings may suggest that studies examining non-survival outcomes, relating to quality of life and supportive care, may benefit from the application of more cautious protocols that make use of remote interventions and measurements. Such an approach might facilitate the recruitment of participants who otherwise may perceive the potential risks to themselves as not outweighed by the broader benefits of the study. Interestingly in our study, hesitancy to participate during the pandemic was confined to participants who had taken part in research studies before; all research naïve participants we interviewed expressed no substantial concerns about participating during the pandemic. We consider this an interesting observation, but the limited scope of our methodology was unable to confirm this as a consistent phenomenon.

A limitation of this study was that we only spoke to UK-based individuals, who commonly possess a cultural affection for the UK's National Health Service (NHS) which may have affected the results, particularly regarding their relationships to their healthcare professionals. A further limitation was that the present study did not explore differences between specific cancer types, or ethnicity. Our sample was limited in the sense that it did not include any minority ethnic individuals, and the online interview method may have introduced an element of digital exclusion. Future studies may wish to examine these issues further, particularly because (1) there were some cancer type-specific issues that were documented in this study (e.g., regarding rare cancer types; or the implications of having brain metastases for travel); and (2) COVID-19 may be exacerbating issues of accessibility to research studies that have intersectional implications regarding health inequalities.

## Conclusions

Individual attitudes towards taking part in a research study during the COVID-19 pandemic vary. Some cancer patients are relatively unconcerned by the pandemic and would be likely to take part in research regardless, but some cited certain conditions for this. Whereas, other patients are concerned about participating in a study during the pandemic; and may be unlikely to do so unless they are provided with adequate reassurance. Consistent with previous research, our findings indicated that likelihood of cancer research participation during an infectious disease epidemic or pandemic may be a function of a basic perceived cost-benefit analysis, which considers the perceived benefit of taking part, either personally to themselves or to wider society. Hence, our study found that previous findings about patients' attitudes to research participation were reproduced in the context of an infectious disease pandemic, but in idiosyncratic forms consistent with the context. Taken with prior research, our study suggests that the following are essential to patient decision-making to take part in research, with regards to COVID-19 concerns: trust in the relevant clinicians/researchers; familiarity with the study location; provision of detailed information on safety protocols for infectious disease; and, in particular, the availability of safe transport.

## Data Availability Statement

The original contributions presented in the study are included in the article/Supplementary Material, further inquiries can be directed to the corresponding author.

## Ethics Statement

This study involving human participants was reviewed and approved by the Research Ethics Office at King's College London (LRS-19/20-19677). The patients/participants provided their written informed consent to participate in this study.

## Author Contributions

LF, HW, AH, SG, JK, and MV contributed to the conception, design of the study, and the writing of the manuscript. LF, HW, AH, and SG conducted study interviews. LF conducted the study analysis. All authors contributed to the article and approved the submitted version.

## Funding

This work was funded by the Medical Research Council (MRC) (grant reference number: MR/R014043/1).

## Conflict of Interest

The authors declare that the research was conducted in the absence of any commercial or financial relationships that could be construed as a potential conflict of interest.

## Publisher's Note

All claims expressed in this article are solely those of the authors and do not necessarily represent those of their affiliated organizations, or those of the publisher, the editors and the reviewers. Any product that may be evaluated in this article, or claim that may be made by its manufacturer, is not guaranteed or endorsed by the publisher.

## Footnotes

^1^Fox L, Beyer K, Rammant E, Morcom E, Van Hemelrijck M, Sullivan R, et al. Impact of the COVID-19 pandemic on cancer research in 2020: a qualitative study of events to inform mitigation strategies. *Front Public Health*. (under review).
